# Biorthonormal Formalism for Nonadiabatic Coupled Cluster Dynamics

**DOI:** 10.1021/acs.jctc.0c00730

**Published:** 2020-12-18

**Authors:** Eirik F. Kjønstad, Henrik Koch

**Affiliations:** † Department of Chemistry, Norwegian University of Science and Technology, Trondheim 7491, Norway; ‡ Scuola Normale Superiore, Piazza dei Cavalieri, 7, Pisa PI 56126, Italy

## Abstract

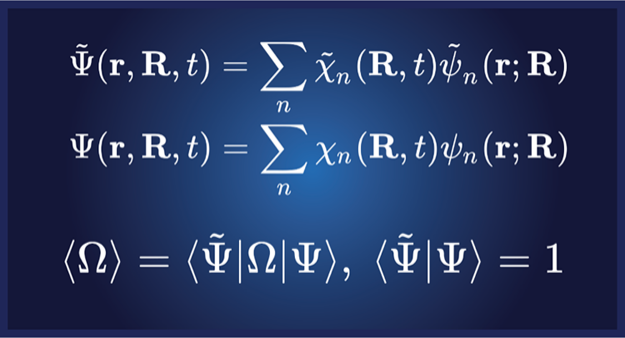

In coupled cluster theory, the electronic states are biorthonormal in the sense that the left states are orthonormal to the right states. Here, we present an extension of this formalism to a left and right total molecular wave function. Starting from left and right Born–Huang expansions, we derive projected Schrödinger equations for the left and right nuclear wave functions. Observables may be extracted from the resulting wave function pair using standard expressions. The formalism is shown to be invariant under electronic basis transformations, such as normalization of the electronic states. Consequently, the nonadiabatic coupling elements can be expressed with biorthonormal electronic wave functions. Calculating normalization factors that scale as full configuration interaction is not necessary, contrary to claims in the literature. For nonadiabatic nuclear dynamics, we need expressions for the derivative couplings in the biorthonormal formalism. These are derived in a Lagrangian framework.

## Introduction

Nonadiabatic coupling elements account for electron–nucleus interactions that are neglected in the Born–Oppenheimer^1^ (BO) approximation. These elements couple different electronic states through the nuclear kinetic energy operator. While mostly negligible in ground-state chemistry, coupling elements are required when considering molecular dynamics in excited electronic states. Excited-state dynamics often involves regions of nuclear space where electronic states are nearly or exactly degenerate, causing a breakdown of the BO separation.^2,3^ Accurately describing nonadiabatic coupling elements is therefore important for reliable predictions in photochemistry.

Coupled cluster theory is one of the most accurate electronic structure methods, both for ground- and excited-state properties,^4−7^ but it has not found widespread use for predicting nonadiabatic dynamics. This is primarily because standard coupled cluster methods give a nonphysical description of regions close to electronic degeneracies or conical intersections.^8−10^ This issue can be traced to the method’s non-Hermiticity, which seems to imply that coupled cluster methods cannot be used for nonadiabatic dynamics. However, this is not the case. As we have shown in recent work, the method can be constrained to give a correct physical description of excited-state conical intersections while retaining the standard non-Hermitian formalism and presumably its accuracy.^11,12^ These developments may lead to renewed interest in simulations of nonadiabatic dynamics that use coupled cluster theory to describe the electronic structure. While ground-state intersections are not easily treated, the method is expected to accurately describe relaxation between excited states.

Nonadiabaticity, as described by coupled cluster methods, has been considered by several authors. The first-order derivative coupling (or vector coupling) was first derived by Christiansen,^13^ who applied the *Z*-vector substitution method^14^ on a biorthonormal expression for the coupling,
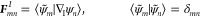
1where (ψ̃_*k*_,ψ_*k*_) refers to the left and right *k*th electronic states and *I* refers to a nucleus. However, Christiansen’s paper^13^ did not include an implementation of the coupling. The vector coupling was later rederived by Tajti and Szalay^15^ by differentiating the corresponding *m*-to-*n* transition element of the electronic Hamiltonian. Their derivation is closely related to that given by Ichino *et al.*(16) for the quasidiabatic interstate coupling. Tajti and Szalay^15^ also gave an implementation at the coupled cluster singles and doubles (CCSD) level.^17^ These papers on the vector coupling^13,15^ did not include a discussion of the nuclear Schrödinger equations in coupled cluster theory, where the coupling elements enter.

The correct formula for the vector coupling has been a subject of some controversy. Tajti and Szalay^15^ argued that the biorthonormal formula in eq 1 is incorrect. As they correctly noted, the vector coupling changes with the norm of the left and right states. A similar observation had been made in an earlier paper on the diagonal BO correction.^18^ Since the vector coupling varies with the norm of the states, the full-CC vector coupling, as given by eq 1, is different from the full configuration interaction (full-CI) limit, where the left and right states are identical and usually normalized. They therefore suggested that normalizing the states was necessary. Furthermore, since the derivative can either act on the left or right state, they suggested using an average of the two.^15^ If true, these observations are troubling: because of the normalization factors for the right states, they imply that computing the vector coupling has a computational cost that scales as full-CI. In practice, the normalization factors are therefore approximated. However, it is unfortunate if one must resort to approximations other than the truncation level of the coupled cluster method (*e.g.*, singles and doubles in CCSD). The need for normalization factors was also assumed in the recent CCSD implementation by Faraji *et al.*(19)

The first main objective of the present paper is to establish that normalization is not necessary. The reason is that normalization is a special case of an invertible transformation of the electronic basis. Such transformations do not change the expansion space in the Born–Huang expansion^20^ and therefore do not change the molecular wave function. In particular, the coefficients in the Born-Huang expansion—that is, the nuclear wave functions—absorb the transformation of the electronic states. The vector coupling does depend on normalization, but this quantity is not an observable. Since normalization is not necessary, the biorthonormal formula in eq 1 is a valid option.

In a recent paper, Shamasundar^21^ found, for the rovibrational Schrödinger equation, that normalization does not affect the Born–Oppenheimer product wave function. He noted that this finding should also generalize to nonadiabatic dynamics, that is, the predicted dynamics should not depend on the normalization of the underlying electronic wave functions.^21^

Derivative couplings are readily derived with the Lagrangian approach.^6,22−24^ Here, we use the Lagrangian introduced for CASCI by Hohenstein^25^ to derive ground-to-excited and excited-to-excited state couplings. This Lagrangian is based on an overlap whose geometrical derivatives are nonadiabatic coupling elements;^25^ the approach bears some similarities to that used by Hättig *et al.*,^26,27^ who derived transition moments as derivatives of transition moment Lagrangians. We use the Lagrangian approach^25^ to derive expressions for the vector coupling as well as the second-order derivative coupling (or scalar coupling)
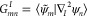
2The scalar coupling is often omitted in dynamics simulations, but its potential influence on nonadiabatic dynamics has been considered in recent years.^3^

The second main objective of the paper is to give a framework for nonadiabatic dynamics using coupled cluster methods. In particular, we argue that the biorthonormal formalism for electronic wave functions implies a biorthonormal formalism for the molecular wave function. Hence, we must determine the left and right nuclear wave functions, and the nuclear motion is described by two sets of nuclear Schrödinger equations. The result is a molecular wave function pair (Ψ̃,Ψ). Observable quantities are calculated by the usual biorthonormal formulas.

## Theory

### Electronic Wave Functions in Coupled Cluster Theory

In the equation of motion coupled cluster formalism, the *n*th (*n* = 0, 1, 2, ...) left and right electronic states are expressed as^28^
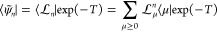
3and
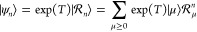
4where ⟨_*m*_ = δ_*mn*_. The projection space is defined as
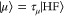
5
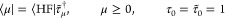
6where τ_μ_ and _μ_ with μ > 0 are the excitation operators relative to the Hartree–Fock state  and ⟨μ|ν⟩ = δ_μν_. The cluster operator is defined as 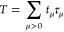
7where {*t*_μ_} are scalars called cluster amplitudes.

The right ground state is assumed to have the exponential form
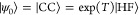
8and projecting the Schrödinger equation onto {⟨μ|}_μ≥0_ gives the ground state equations
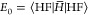
9
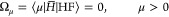
10where *H̅* = exp(−*T*)*H* exp(*T*). The state amplitudes,  and , are determined by making the *n*th energy stationary under the condition . The result is a set of eigenvalue equations
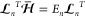
11
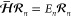
12where *E*_*n*_ is the energy of the *n*th state and . Here and throughout, we use bold font (***X***) to denote vectors (*X*_μ_) and matrices (*X*_μν_). In summary, eqs 10–12 are solved to determine the left and right electronic states. The reader is referred to the literature for more details.^28,29^

### Born–Huang Expansion of the Total Wave Function and the Nuclear Schrödinger Equations

The Born–Huang expansion expresses the total wave function in terms of the electronic wave functions. The coefficients of the expansion define the nuclear wave functions. These nuclear wave functions are determined by inserting the expansion in the Schrödinger equation and projecting out the electronic components; the result is a set of nuclear Schrödinger equations. In coupled cluster theory, this implies a biorthonormal description of the total wave function since we can expand in both the left and right electronic wave functions (_*n*_ and ψ_*n*_). Hence, we have a left and a right total wave function given by the Born–Huang expansions
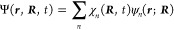
13
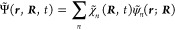
14with associated left and right nuclear wave functions χ̃_*n*_ and χ_*n*_, and
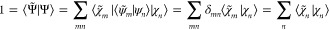
15where we have assumed biorthonormal electronic states in the third equality:  = δ_*mn*_. In eqs 13 and 14, the electronic and nuclear coordinates are denoted by ***r*** and ***R***, respectively, and time by *t*. Expectation values are defined through the standard expression^28,29^
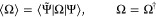
16

To derive the equations for the nuclear wave functions, one normally projects the total Schrödinger equation on the electronic basis. In this respect, a biorthonormal description is advantageous; for practical coupled cluster models, where the excitation space is truncated to some excitation order, projection of the right Schrödinger equation is done onto the left electronic basis, leading to computationally tractable expressions that scale as expected for the given model (*e.g.*,  for CCSD, where *N* is the size of the system).

By inserting the Ψ in eq 13 into the time-dependent Schrödinger equation
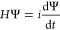
17and projecting it onto the left electronic basis, we get a coupled set of equations for the right nuclear wave functions χ_*n*_. These nuclear Schrödinger equations can be expressed as
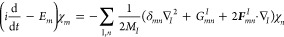
18where we have suppressed the ***R*** and *t* dependence for readability. The nonadiabatic coupling vectors in eq 18 are given in the biorthonormal basis
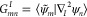
19
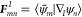
20

In analogous fashion, we derive the nuclear Schrödinger equations for the left nuclear wave functions from the complex conjugated Schrödinger equation
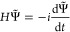
21

Inserting eq 14 into 21 and projecting onto the right electronic basis leads to
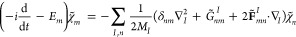
22
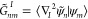
23
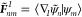
24

The nuclear Schrödinger equations can be expressed in the more compact matrix notation
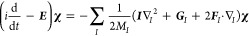
25
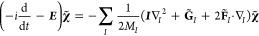
26where ***E*** is a diagonal matrix with the electronic energies on the diagonal, ***I*** is the identity matrix, **χ** is a vector containing the right nuclear wave functions, and ***G***_*I*_ and ***F***_*I*_ are matrices consisting of the scalar and vector couplings of the *I*th nucleus, respectively. The quantities with a tilde are similarly defined.

This matrix notation has been used to illuminate some relations to gauge theories in the nuclear Schrödinger equations; Pacher *et al.*(30) found that the vector coupling can be seen to serve a role analogous to the vector potential in electromagnetism. In the present work, it serves as a useful notation for dealing with basis transformations and the vector algebra needed to demonstrate invariance under such transformations.

### Basis Invariance and the Special Case of Norm Invariance

It has been suggested that normalizing the electronic states is necessary when calculating nonadiabatic coupling elements.^15^ The reason is that the left and right states are biorthonormal in the coupled cluster theory. Compared to the couplings in the full-CI theory, where the states are normalized, the full coupled cluster limit is “incorrect” because the couplings depend on the geometry-dependent normalization constants. While this suggests that one should normalize the states, doing so is not feasible. The computational cost of the normalization factor scales as full-CI for the right electronic states^15^
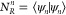
27
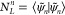
28Since one cannot evaluate *N*_*R*_^*n*^ in general, some have suggested *N*_*R*_^*n*^ = (*N*_*L*_^*n*^)^−1^ or *N*_*R*_^*n*^ = *N*_*L*_^*n*^ = 1 as alternatives. The former gives the full-CI limit, while the latter is equivalent to assuming the standard biorthonormality.^15,19^

Biorthonormality is not an issue from the point of view of nonadiabatic dynamics. In fact, normalizing the electronic states is a special case of basis transformation of the electronic basis. As such, the Born–Huang expansion and the nuclear Schrödinger equations are equivalent in the transformed and untransformed bases. Changes in the electronic basis are absorbed in the expansion coefficients, that is, the nuclear wave functions. In the case of normalization, the right electronic wave functions are divided by *N*_*R*_^*n*^, while the right nuclear wave functions are multiplied by *N*_*R*_^*n*^. The total wave function does not change.

More precisely, consider invertible transformations of the left and right electronic bases. In vector notation, these transformations can be expressed as
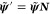
29
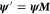
30where the matrices ***M*** and ***N*** are assumed to be smooth invertible matrix functions of the nuclear coordinates. For notational simplicity, we have let the left and right wave function vectors be row vectors. Transformed quantities are denoted by a prime. In the transformed basis, the total left and right wave functions have the Born–Huang expansions
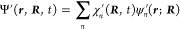
31
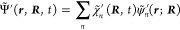
32

We wish to show that the wave function in the transformed basis is identical to that obtained in the untransformed basis, that is, Ψ′ = Ψ and Ψ̃′ = Ψ̃. The conclusion that follows is that the choice of electronic basis does not change the predictions of the theory. In other words, it is perfectly appropriate to use the standard^28,29^ biorthonormal description.

Before proceeding, we define some notation. In the transformed basis, we have to account for the nonunit overlap of the electronic wave functions. Hence, when projecting the time-dependent Schrödinger equation onto the electronic basis, we get electronic overlap matrix elements. In particular
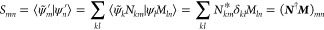
33Similarly, the electronic Hamiltonian matrix is not necessarily diagonal


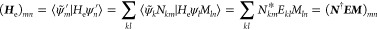
34

We show the equivalence for the right wave functions. The proof for the left wave function is identical. Following the standard procedure, we now insert the transformed wave function in eq 31 into the Schrödinger equation and project onto the transformed left electronic wave functions. The result is the right nuclear Schrödinger equation
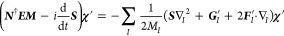
35If the total wave function is invariant and **ψ**′ = **ψ*M***, then we must have nuclear wave functions that cancel the transformation of the electronic wave functions, meaning that


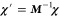
36Indeed, with **χ**′ as given in eq 36, we have


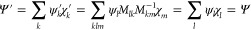
37

Let us confirm that eq 36 is in fact a solution to the transformed nuclear Schrödinger equation in eq 35. We begin by relating the old and new nonadiabatic coupling terms. The gradients of the electronic wave functions transform as
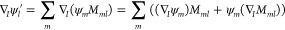
38Hence, the vector couplings can be written as


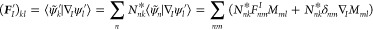
39In more compact matrix notation, we have


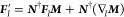
40Similarly, the Laplacian of the electronic wave functions transforms as
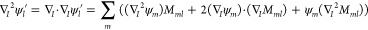
41implying that the scalar couplings transform as
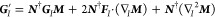
42

The gradient and Laplacian of **χ**′ is derived in the same way as for the electronic states, giving
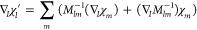
43
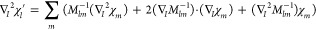
44Thus, we have the following contributions on the right-hand side of the nuclear Schrödinger equation


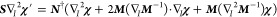
45
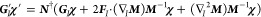
46
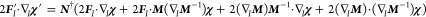
47Though somewhat involved, most of the terms cancel when added together. In fact, since


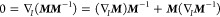
48
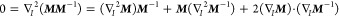
49we can write
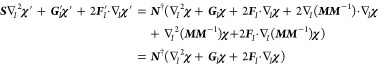
50In other words, with **χ**′ = ***M***^–1^**χ**, the right nuclear Schrödinger equation simplifies to


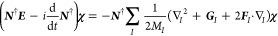
51which, upon premultiplication by ***N***^–†^, is seen to be equivalent to the original right nuclear Schrödinger equation in eq 25.

Since all of the steps we have made are reversible, we have shown that **χ** is a solution to the untransformed nuclear Schrödinger equation if and only if **χ**′ is a solution to the transformed Schrödinger equation. The total right wave function is therefore invariant with respect to transformations of the electronic basis, Ψ′ = Ψ. One consequence of basis invariance is that the nonadiabatic couplings can be expressed in the standard biorthonormal formalism.

In the next section, we derive these in a Lagrangian framework. The couplings have contributions that arise from the geometry-dependence of the many-body operators, where a choice of orbital connection is necessary.^13,31^ These terms are described in Appendix A.

### Nonadiabatic Coupled Cluster Couplings in a Lagrangian Formalism

To obtain a Lagrangian for the vector coupling in CASCI, Hohenstein^25^ defined a partially frozen overlap whose first derivatives are identical to components of the vector coupling. In coupled cluster methods, this overlap can be expressed as
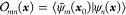
52in terms of which we have
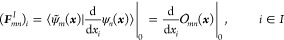
53and
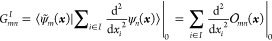
54We let *i* ∈ *I* mean that *x*_*i*_ is one of the three coordinates of the *I*th nucleus (*x*, *y*, or *z*). Note that  depends on ***x***_0_. We suppress this dependency to keep the notation simple.

The overlap is expressed in terms of coupled cluster wave functions, which depend not only on ***x*** but also on a set of wave function parameters **λ** (which themselves depend on ***x***). Written out in terms of the parameters, we have
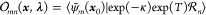
55where
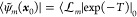
56and
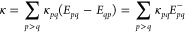
57

The κ operator accounts for orbital rotations, meaning changes in the Hartree–Fock orbitals, where, by definition, we have κ(***x***_0_) = 0. Following the standard recipe, we add the equations (denoted by ) that determine the parameters as constraints with associated Lagrangian multipliers (denoted by **γ**)
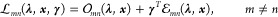
58where **λ** and **γ** are determined for every ***x*** by the stationarity conditions
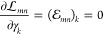
59
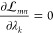
60

The derivatives of this Lagrangian are identical to the derivatives of the frozen overlap (since ). One advantage of the Lagrangian formalism is that it automatically incorporates the 2*n* + 1 and 2*n* + 2 rules for **λ** and **γ**, respectively.^6^ In particular, we have
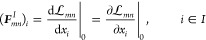
61where the final equality follows from stationarity, see eqs 59 and 60. Denoting partial derivatives with respect to geometrical coordinates as
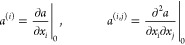
62we can write
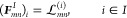
63

Furthermore, if we let
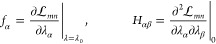
64then the scalar coupling can be expressed as (see Appendix B)
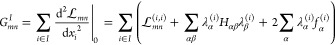
65Clearly, ***F***_*mn*_^*I*^ and *G*_*mn*_^*I*^ are similar in complexity to the energy gradient and Hessian. However, *G*_*mn*_^*I*^ is somewhat simpler than the energy Hessian because the first derivatives of the parameters (**λ**^(*i*)^) can be considered one at a time.

To proceed, we must define the Lagrangian  in detail. The conditions  include all equations that must be solved to evaluate the overlap . These are (a) the Hartree–Fock equations, (b) the amplitude equations, and (c) the eigenvalue equations for the right state amplitudes. Written out in full, we have
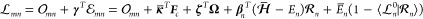
66where we have introduced multipliers associated with the different sets of equations, **κ̅**, **ζ**, as well as β_*n*_ and *E̅*_*n*_. We have also introduced the Brillouin condition
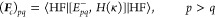
67where
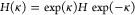
68Furthermore, the similarity transformed the Hamiltonian in **Ω** and  is given by


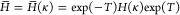
69and the *n*th electronic energy is defined as
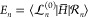
70

With  defined, we can now consider the equations for the zeroth order multipliers. These are determined from the zeroth order terms of the **λ** stationarity, eq 60. To keep our notation simple, we will denote the zeroth order terms as **γ**^(0)^ ≡ **γ** and **λ**^(0)^ ≡ **λ**, where it should be understood from context when these are **γ** and **λ** evaluated at ***x***_0_. Differentiation with respect to the state parameters gives
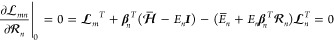
71To solve this equation, we note that if we let


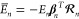
72the equation for **β**_*n*_ becomes
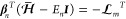
73Thus, we have


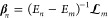
74

Next, we consider stationarity with respect to ***t***. This can be expressed as
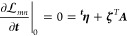
75where
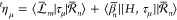
76and where we have introduced the notation
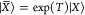
77
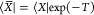
78

Finally, we have stationarity with respect to **κ**, which can be written as
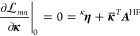
79where
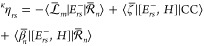
80and
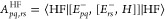
81

With the zeroth order multipliers determined, we can derive the expression for the vector coupling. By partially differentiating , we find that
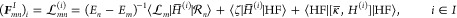
82where
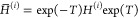
83and where quantities at ***x***_0_ are denoted as *y*^(0)^ ≡ *y* (*e.g.*, we denote *T*^(0)^ as *T*). Here, we have assumed the natural connection, for which there are no contributions to ***F***_*mn*_^*I*^ that originate from the many-body operators (see Appendix A and refs (13) and (31)).

The vector coupling given in eq 82 has also been identified by other authors. It was derived by Christiansen,^13^ who assumed biorthonormality and used *Z*-vector substitution^14^ on the expression for the vector coupling. Tajti and Szalay^15^ identified the same expression indirectly using *Z*-vector substitution on derivatives of Hamiltonian transition elements. However, they also argued^15^ that the coupling should not be given by eq 82 but rather be averaged and expressed with normalized states. As we have shown, eq 82 is a valid choice due to norm invariance and represents the vector coupling in the right nuclear Schrödinger equations. For the left Schrödinger equations, we can make use of the identity
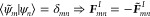
84

Before moving on to the scalar coupling, we note that although the *Z*-vector substitution method is equivalent to the Lagrangian technique, the latter method gives, in our opinion, an especially elegant way of deriving the coupling elements.

For the scalar coupling, we must determine the first derivatives of the parameters. Equations for these are obtained as the first-order terms of the multiplier stationarity conditions. In the case of ***t***, we have
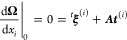
85where
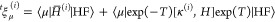
86In the case of **κ**, we similarly have


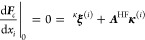
87where
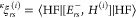
88The biorthonormality condition implies


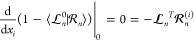
89while the eigenvalue condition implies
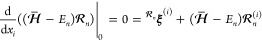
90Here, we have defined
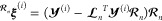
91where
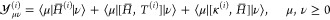
92

With the derivatives of the parameters determined, let us next consider *f*_α_^(*i*)^ and *H*_αβ_, see eq 64. Recall that the α and β indices refer to the parameters λ_α_ and λ_β_. The gradient ***f*** is given by the zeroth order equations for the multipliers, that is, eqs 71, 75, and 79, with **λ** = **λ**_0_ but allowing for ***x*** ≠ ***x***_0_. Partially differentiating these terms with respect to *x*_*i*_ gives ***f***^(*i*)^. The blocks of the ∑_α_*f*_α_^(*i*)^λ_α_^(*i*)^ contributions to *G*_*mn*_^*I*^ can be written
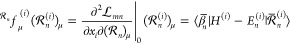
93and
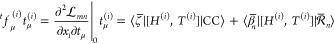
94
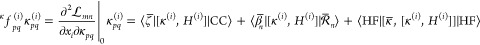
95where repeated indices implies summation. For contributions to *G*_*mn*_^*I*^ involving *H*_αβ_ we have, for terms involving ***t*** and **κ**,
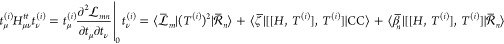
96as well as
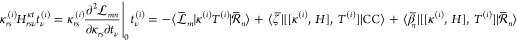
97and
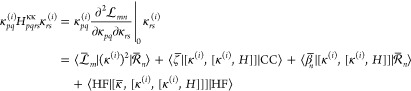
98

Next, we have terms involving right state and the cluster amplitudes and orbital rotations, *i.e.*
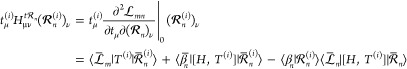
99
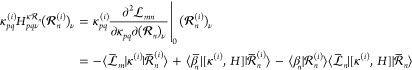
100Finally, we have the partial derivative of the Lagrangian, which can be written as


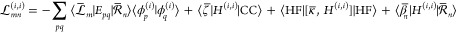
101

Written in compact notation, the scalar coupling is
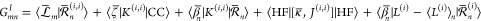
102where we have let
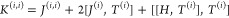
103
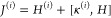
104
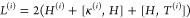
105
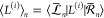
106
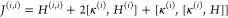
107as well as
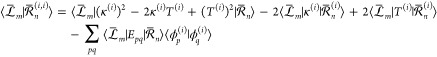
108The final term in eq 108 arises from the orbital connection (see Appendix A).

Throughout the derivations above, we have considered the off-diagonal coupling elements (*m* ≠ *n*). The diagonal terms can be derived from the Lagrangian
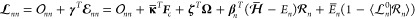
109which gives the slightly different  stationarity condition
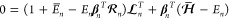
110Here, we again select *E̅*_*n*_ to make the first term vanish, giving
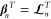
111

Other than this change, the derivation of the scalar coupling is virtually unchanged. Terms involving differentiation of  has the left state  in the bracket instead of  (*e.g.*, in the stationarity conditions for the zeroth order multipliers). In particular, the expression in eq 102 is valid with *m* = *n*.

Unlike for the vector coupling, there is no convenient relationship between *G*_*mn*_^*I*^ and *G̃*_*mn*_^*I*^. To derive the latter quantity, we may consider the Lagrangian
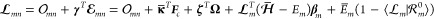
112where
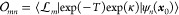
113
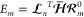
114The  stationarity then gives


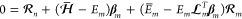
115from which we again have  and thus
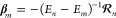
116

The equations for the zeroth order multipliers are derived as before, with the result that the multipliers change their sign, thus giving the result in eq 84 for the vector coupling. For the derivative of the parameters, we have the same equations for ***t***^(*i*)^ and **κ**^(*i*)^. For the derivative of , we must solve the equation
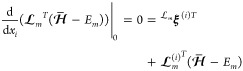
117which is analogous to eq 90. In contributions involving  in *G*_*mn*_^*I*^, we obtain similar expressions involving  in the case of *G̃*_*mn*_^*I*^. The end result is
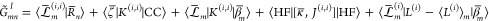
118with
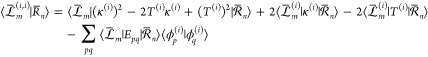
119

Finally, *G̃*_*nn*_^*I*^ is obtained in a manner similar to *G*_*nn*_^*I*^, see eq 109 and the surrounding text.

This concludes our derivation of the coupled cluster scalar coupling. To the best of our knowledge, equations for this coupling (with *m* ≠ *n*) have not been presented in the literature before. Diagonal terms were also considered by Gauss *et al.*(18) from a different starting point.

## Concluding Remarks

The norm of the electronic states changes the value of nonadiabatic coupling elements but does not change the molecular wave function. The biorthonormal formula assumed by Christiansen^13^ is therefore a valid choice for nonadiabatic dynamics using coupled cluster methods. More generally, we have shown that the total wave function is invariant with respect to smooth and invertible transformations of the electronic basis. Of course, the biorthonormal couplings are not directly comparable to the coupling elements of an Hermitian method with normalized states, such as CI or full-CI. However, this reflects the basis-dependence of the couplings and not the validity of the biorthonormal formalism.

We therefore derive a set of nuclear Schrödinger equations assuming biorthonormal projection onto the electronic basis. Combined with expressions derived for the vector and scalar couplings, these nuclear Schrödinger equations serve as a starting point for the application of coupled cluster methods in simulations of nonadiabatic dynamics.

Our derivations have been restricted to the standard coupled cluster theory. However, the Lagrangian formalism is easily extended to similarity constrained coupled cluster methods,^11,12^ which are suited to describe relaxation through a conical intersection between excited states. The application to ground-state intersections is less straightforward but may be accessible with approaches that use a different reference than the closed-shell Hartree–Fock state.^32^

## Appendix A

### Geometry Dependence of the Many-Body Operators

The scalar and vector couplings, see eqs 19 and 20, involve differentiation of the electronic wave functions with respect to the nuclear coordinates ***x***. To evaluate these, we need to consider the dependence of both the wave function parameters and the many-body operators. For reasons that will become clear, we use the so-called natural connection^31^ to describe the orbital basis at neighboring geometries. Our presentation follows closely that given by of Olsen *et al.*(31) Using the natural connection for nonadiabatic coupling elements was also discussed by Christiansen.^13^

To evaluate derivatives at ***x***_0_, we relate the basis at ***x***_0_ to some basis at ***x*** = ***x***_0_ + Δ***x***. Suppose the molecular orbitals (MOs) at ***x***_0_ are

120

where *C*_α*m*_ are orbital coefficients and χ_α_ are atomic orbitals. The unmodified MOs (UMOs) are defined by freezing the orbital coefficients

121

The UMOs are not orthonormal, however

122

Hence, UMOs are related to orthonormalized MOs (OMOs) through

123

where the connection matrix ***T***(***x***) satisfies ***T***(***x***_0_) = ***I*** and ***T***(***x***)^†^***S***(***x***)***T***(***x***) = ***I***.

In the natural connection, ***T*** is chosen to be

124

where

125

Let us now relate the orbital space at ***x*** to the one at ***x***_0_. In order to do so, we consider a complete orbital basis (denoted by indices *pq*...) partitioned into the OMO basis (*mn*...) and the orthogonal complement orbitals or OCOs (*uv*...). For complete bases, we can write

126

Occupation number states at ***x*** can thus be expressed as

127

with

128

where *b*(***x***) is the anti-Hermitian operator with *b*_*pq*_(***x***) defined such that ***U***(***x***) = exp(−***b***(***x***)). The many-body operators can be expanded as

129

To evaluate derivatives with respect to some specific component (denoted *x*), we consider the Taylor expansion of a displacement in this direction, ***x*** = ***x***_0_ + Δ***x***, which can be expressed as

130

131

where *b*^(*n*)^=∑_*pq*_*b*_*pq*_^(*n*)^*a*_*p*_^†^*a*_*q*_. Here, we have let *a*_*p*_^†^ ≡ *a*_*p*_^†^(***x***_0_) and suppressed the ***x***_0_-dependence of the derivatives. It is useful to split operator contributions in the OMO (∥) and OCO (⊥) blocks

132

Now, we can evaluate the first derivative contribution

133

Using eq 127, we get

134

To simplify further, we note that *U*_*mn*_ = Δ_*mn*_ is Hermitian in the natural connection. Since the *U*_*uv*_ block can similarly be chosen to be Hermitian, we have^31^

135

and so

136

In general, *f*_*IJ*_ is non-zero with connections other than the natural connection.

Next, we evaluate the second derivative contribution

137

which can be written as

138

In the final equality, we have used eq 135. Now, notice that since

139

the only non-zero *b*^(1)^*b*^(1)^ contribution is the one that first creates an electron in the complementary space and then destroys it. Thus

140

The commutator can be expressed as

141

Moreover, since

142

the inner projection in eq 141 is equivalent to the identity and so

143

Hence

144

The formulas for *f*_*IJ*_ and *g*_*IJ*_ are valid for occupation number states but allow for generalization to general wave functions. We will be concerned with evaluating partial derivatives with respect to *x* for wave functions of the form

145

Since *c*_*Ik*_ depends implicitly on ***x***, we have ∂*c*_*Ik*_/∂*x* = 0. Thus

146

and

147

For partial derivatives of the energy, we also have to account for the explicit ***x***-dependence of the Hamiltonian. We express the OMO Hamiltonian as

148

where both the integrals and the operators depend on ***x***. However, the dependence of the operators can be ignored in energy derivatives because

149

In particular, elements involving *E*_*pq*_(***x***) and *e*_*pqrs*_(***x***) are linear combinations of such overlaps and therefore give no contributions in energy derivatives.^33^ The integrals are related to the UMO basis as

150

151

By differentiating ***TW*** = ***W***^†^***T***^†^ and ***I*** = ***T***^†^***ST***, we find that ***T***^(1)^ = −***W***^(1)^. Consequently, the partial derivative of the Hamiltonian can be written as

152

where *H*_*u*_^(1)^ is the derivative of the UMO Hamiltonian and

153

where *j*_*pq*_ and *j*_*pqrs*_ are the sums of one-index transformations of *W*_*pq*_^(1)^ with *h*_*pq*_ and *g*_*pqrs*_, respectively.^34^ The ***W***^(1)^ matrix, given by

154

is analogous to *S*_*pq*_^(1)^ = ∂*S*_*pq*_/∂*x* in the symmetric connection ***T*** = ***S***^–1/2^.

This concludes our discussion of how the geometry dependence of the many-body operators affects energy derivatives and nonadiabatic coupling elements. We refer the reader to the literature for more details.^31,33^

## Appendix B

### Lagrangian Derivatives

Here, we derive the first and second derivatives of the generic Lagrangian

155

with respect to ***x***. The parameters and multipliers both depend on ***x*** since they are determined, for a given ***x***, from the stationarity conditions

156

Using Einstein notation, we can write the Taylor expansion of  about some ***x***_0_ as

157

where we have ignored terms of order 3 or higher in Δ***x***. These terms do not contribute to the first and second derivatives and are therefore not relevant to the analysis given here.

In the first derivative, only the partial derivative survives, *i.e.*

158

This is due to the stationarity conditions since they ensure that there are no linear terms in Δ**λ** and Δ**γ** in the Taylor expansion in eq 157. In the second derivative, it is convenient to introduce notation for derivatives with respect to particular components of ***x***. We let

159

160

Then, we can write

161

and

162

Now

163

by stationarity, so that

164

To simplify the notation further, we define derivatives with respect to the parameters as

165

Thus, we get the final expression for the second derivatives:

166
